# Passive stretching decreases muscle efficiency in balance tasks

**DOI:** 10.1371/journal.pone.0256656

**Published:** 2021-09-22

**Authors:** Giuseppe Coratella, Stefano Longo, Susanna Rampichini, Christian Doria, Marta Borrelli, Eloisa Limonta, Giovanni Michielon, Emiliano Cè, Fabio Esposito

**Affiliations:** 1 Department of Biomedical Sciences for Health (SCIBIS), Università degli Studi di Milano, Milan, Italy; 2 IRCCS Galeazzi Orthopedic Institute, Milan, Italy; Toronto Rehabilitation Institute - UHN, CANADA

## Abstract

The current study aimed to verify whether or not passive static stretching affects balance control capacity. Thirty-eight participants (19 women and 19 men) underwent a passive static stretching session, involving the knee extensor/flexor and dorsi/plantarflexor muscles, and a control session (no stretching, CTRL). Before (PRE), immediately after (POST), after 15 (POST_15_) and 30 min (POST_30_) from stretching (or rest in CTRL), balance control was evaluated under static and dynamic conditions, with open/closed eyes, and with/without somatosensory perturbation (foam under the feet). During tests, centre of pressure (CoP) sway area and perimeter and antero-posterior and medio-lateral sway mean speed were computed. Surface electromyography root mean square (sEMG RMS) was calculated from the *vastus lateralis*, *biceps femoris*, *gastrocnemius medialis*, and *tibialis anterior* muscles during MVC and during the balance tests. Hip flexion/extension and dorsi/plantarflexion range of motion (ROM), maximum voluntary contraction (MVC) and sEMG RMS during MVC were measured at the same time points. After stretching, ROM increased (≈6.5%; *P*<0.05), while MVC and sEMG RMS decreased (≈9% and ≈7.5%, respectively; *P*<0.05). Regardless of the testing condition, CoP sway area and the perimeter remained similar, while antero-posterior and medio-lateral sway mean speed decreased by ≈8% and ≈12%, respectively (*P*<0.05). sEMG RMS during the balance tests increased in all muscles in POST (≈7%, *P*<0.05). All variables recovered in POST_30_. No changes occurred in CTRL. Passive static stretching did not affect the overall balance control ability. However, greater muscle activation was required to maintain similar CoP sway, thus suggesting a decrease in muscle efficiency.

## Introduction

Balance control (BC) is the process of maintaining the body centre of gravity vertically over the base of support. It relies on rapid, continuous feedback and integration of afferent information coming from three sensory components, the somatosensory, visual, and vestibular systems, resulting in smooth and coordinated neuromuscular actions [1]. Any external influence on each of the three afferent systems may lead to an improvement or impairment of the motor response aimed at maintaining balance [2]. For example, perturbations in the somatosensory system following ankle sprain [3] or impairments of the vestibular system [2] can reduce BC ability.

Muscle stretching is a method widely used in sport and rehabilitation with the aim of increasing joint range of motion (ROM). However, a passive static stretching (PS) bout was reported to impair acutely the force-generating capacity of the stretched muscle [4, 5] via possible changes in its mechanical (reduction in muscle and/or muscle-tendon unit stiffness) [6, 7] and neuromuscular properties [4]. Particularly, an alteration in the afferent feedback coming from the proprioceptors of the stretched muscle has been advocated as a possible mechanism underlying the PS-induced reduction in muscle force-generating capacity [4, 5]. Interestingly, these mechanical and neuromuscular factors may also affect BC [3, 8, 9], even though the literature on this subject is controversial. Indeed, improvements [10–12], worsening [3, 8, 9], or no changes [10] in BC were reported after PS. On the one hand, BC could be enhanced by a PS-induced improvement in the ability to perceive the joint position [10–12] and by a PS-induced decrease in muscle-tendon unit stiffness, which could reduce the stretch-reflex activity [13]. On the other hand, BC could be impaired by an alteration in the somatosensory feedback and by the greater joint mobility induced by PS, which would imply greater muscle activation to stabilise the joint during BC tasks [3, 8, 9]. Thus, including the assessment of muscle activation during a given balance task may provide information about the behaviour of the stretched muscles involved. Moreover, other variables such as the PS protocol duration, a previous experience in BC training (expert vs novice) [10], and different assessment protocols (e.g., static vs dynamic condition) could likely explain the disparity in the results reported in the literature. Studies aiming to clarify the PS-induced effects on BC under different conditions (static and dynamic) and the possible mechanisms underpinning such changes are therefore needed.

By manipulating the contribution of the afferent systems involved in BC, it would be possible to highlight the compensatory mechanisms played by the other sensory components when one is limited. This may help to clarify which afferent system would be affected by a PS bout, and possibly contribute to explain why discordant data on the effects of PS on BC exist. For example, during closed eyes tests the visual afferences are excluded, forcing to use mainly vestibular and somatosensory information to maintain BC [2]. Similarly, the use of a foam pad positioned under the feet could minimise the somatosensory information coming from the lower limbs, thus forcing to rely mainly on vestibular and visual feedback [2, 14]. Since PS might affect the somatosensory feedback, it is possible that balance tasks where this is prevalent would be affected more than others. As such, impairments in closed eyes tasks would be more pronounced compared to the balance tasks with a foam pad under the feet. This strategy, together with the assessment of lower limb muscle activation mostly involved in BC, could help in providing further insights on the influence of PS on BC.

With this in mind, the aim of the study was to evaluate the acute effects of PS on BC tested under static and dynamic modality. By manipulating the testing variables (i.e., open/closed eyes, with/without foam pad) and by assessing the activation levels of the muscles mostly involved in BC, we hypothesised that after PS, BC would deteriorate in both static and dynamic conditions with an increase in muscle activation level, especially in the closed eyes condition. In contrast, when the somatosensory feedback is limited (i.e., with foam pad under the feet), BC and muscle activation level would be less affected, likely due to a compensation of the visual and vestibular system to the possible stretch-induced somatosensory alterations.

## Materials and methods

### Sample size calculation

For this cross-sectional, within-subject study, sample size calculation was based on previous investigations, considering the stretch-induced changes in BC as the reference parameter [3, 9] and computed using statistical software G-Power 3.1 (Düsseldorf, Germany). From these studies, a Cohen’s *d* effect size (ES) of ~0.15 was obtained. By considering this ES, two-tail effect, α = 0.05, and required power (1 - β) = 0.80, the desired sample size resulted in 30 participants. However, given the procedures and the possible high variability in the recorded signals, 38 participants were recruited to decrease any possible risk of bias.

### Participants

Thirty-eight healthy participants [19 women and 19 men, age 26(3) years; stature 1.73(0.10) m; body mass 69(17) kg; mean(SD)] volunteered for the present study. The participants were recreationally active, with no evident orthopaedic and/or neurological pathologies, no lower-limb muscular or joint injuries in the previous 6 months, and not involved in a systematic PS programme in the previous 6 months. The Ethics Committee of the Università degli Studi di Milano approved the study (CE 23/20), which was performed in accordance with the principles of the latest version of the Declaration of Helsinki. The participants gave their written, informed consent after receiving an explanation of the purpose of the study and the experimental procedures. The participants were free to withdraw from the study at any time. During the experimental procedures, they were asked to abstain from alcohol, caffeine or similar beverages in the 24 h preceding the test, as well as to refrain from any form of vigorous physical activity. All the experiments were performed following the safety procedure for exercise testing in the scenario of COVID-19 [15].

### Experimental design

All measurements and the stretching protocol were conducted in a laboratory with constant temperature and relative humidity [22(1)°C and 50(5)%, respectively]. To limit possible bias induced by the circadian rhythm of the measurements, all tests were repeated at the same time of day. The entire protocol lasted a total of four weeks. As all participants had never been involved in BC testing and training protocols and being the learning effect a possible bias in the study, the first three weeks were devoted to the familiarization phase. During this period, six sessions were scheduled within three weeks with at least 72 h in between. In each session the participants became familiar with the tests for the determination of the maximum voluntary isometric contraction (MVC) and with the BC tests in static and dynamic conditions, with all the variants proposed in the study. From the analysis of the results from the various balance tests, it was found that six sessions were sufficient to reach a plateau condition, indicating a good control of the execution of the tests. After the familiarization phase, all participants underwent two experimental sessions, stretching (STR) and control (CTRL), proposed in random order with at least 72 h of pause in between. Considering the influence of the menstrual cycle on BC [16], the women involved in the study performed all the tests during the same menstrual cycle period. In these sessions, the hip extension/flexion and ankle dorsi/plantarflexion maximum ROM were assessed on the dominant lower limb (the limb used to kick a ball). The MVC of the knee extensor/flexor muscles and ankle dorsi/plantarflexor muscles was then determined. Thereafter, the participants performed the bipedal BC tests in static and dynamic modality under different conditions: open eyes (OE), closed eyes (CE), without, and with foam pad (+foam). The order of all tests was randomised. ROM, MVC and BC tests were conducted by a single operator each. During the ROM, MVC, and BC tests, the surface electromyographic (sEMG) signal was detected in the *vastus lateralis*, *biceps femoris*, *gastrocnemius medialis*, and *tibialis anterior* of the dominant limb. All the tests were performed before (PRE), immediately after (POST) and after 15 (POST_15_) and 30 min (POST_30_) from the execution of the stretching in STR, or after an equivalent period during which the participant remained laying down on a medical bed in CTRL.

### Measurements and data analysis

The operators who analysed the data were blinded to the condition (STR or CTRL).

#### ROM

A biaxial angle transducer (mod. TSD130B; Biopac System, Inc., Santa Barbara, CA, USA) was used for all measurements. The angle transducer signals were driven to an A/D converter (mod. UM 150, Biopac System Inc., Santa Barbara, CA, USA), sampled at 1000 Hz, directed to an auxiliary input of the electromyography amplifier (mod. EMG-USB, OtBioelettronica, Turin, Italy) and stored on a personal computer. For hip ROM, the fixed axis was positioned at femoral head level parallel to the trunk, while the movable axis was positioned on the proximal one third of the femur, parallel to the femoral shaft axis. For ankle ROM, the fixed axis was positioned superior to the external malleolus, parallel to the axis of the fibular shaft, and the mobile axis parallel to the calcaneus. During the measurements the participants remained supine with the feet outside the medical bed, except for the hip extension measurement, during which they were in a prone position. The participants were instructed to remain passive during the assessment, and the measurement stopped when the perceived discomfort point was reached, i.e., when the participants were not able to further tolerate the muscle elongation without perceiving pain. To avoid reflex muscle activations during the test, the movement was performed slowly in 6 s marked by a metronome. The sEMG signal of the muscles involved in the manoeuvre was checked to monitor possible muscle activation during elongation. If the sEMG signal was >5% of that obtained during the MVC, ROM assessment was repeated. Three trials were performed. The maximum angle reached in each set was measured to calculate the maximum ROM. All measurements were performed by the same operator three times for each test interspersed by 1 min of rest. The maximum value reached was considered as ROM.

#### MVC

The knee extensor/flexor muscle and the ankle dorsi/plantarflexor muscles MVC were measured following the procedures used in previous studies [17, 18]. Briefly, for the assessment of knee extensor/flexor and ankle dorsiflexor muscles, the participants were seated on an ergometer with the trunk erect, and with the hip and the ankle flexed at 90°. The trunk was secured to the seat with inelastic bands adjusted by Velcro® straps. The knee joint was positioned at 90° during the tests for the knee extensor and dorsiflexor muscles, while it was set at 60° (0° = full knee extension) for knee flexor muscles testing. The plantarflexor muscles were tested in prone position, with the ankle positioned at 90° (i.e., neutral position) and inserted into an immovable support. The shoulders were constrained by supports so that trunk movements were minimised during the contraction phase. In all tests a load cell (mod. SM-2000 N operating linearly between 0 and 2000 N; Interface, Crowthorne, UK) was used for the detection of the force signal. The load cell was attached to the ankle in knee extensor/flexor muscles test, and to a metal plate under the foot in the dorsi/plantarflexor muscles test. The force signal was driven to an A/D converter (mod. UM 150, Biopac, Biopac System Inc., Santa Barbara, CA, USA), sampled at 1000 Hz, directed to an auxiliary input of the electromyography amplifier (mod. EMG-USB, OtBioelettronica, Turin, Italy) and stored on a personal computer. After a standardized warm-up (10 x 2-s contractions at 50% MVC determined during familiarization), three contractions were performed for each muscle in PRE, and a single contraction in POST, POST_15_, and POST_30_. Each contraction lasted 3 s with a 2-min pause between contractions. The maximum value reached in the different contractions was identified as MVC.

#### BC tests

BC tests were conducted under static and dynamic conditions on a computerised stabilometry platform (Prokin 252, Tecnobody, Bergamo, Italia). The platform consisted of three strain gauges set in a triangular position under a surface of 55 cm in diameter with a 40-Hz sampling rate and a sensitivity of 0.1 [19]. The operator was blinded about the participants’ experimental session (STR or CTRL). The total duration of BC tests was 10 min (5 min for static and 5 min for dynamic tests). To increase the level of concentration and motivation, the participants were tested alone in a separate room close to the laboratory. All tests were conducted barefoot. The feet position was the same in both STR and CTRL testing sessions.

In OE static balance tests, the participants stood upright with a bipedal stance while visualising a marker shown on a screen in front of them. Following the manufacturer’s indications, the stance was standardised as follows: internal malleoli distance of 5 cm and foot axis tilted at 30° in respect to the sagittal plane. The screen height was tailored to provide each participant with a clear screen view without any cervical spine flexion or extension. The knees were extended, and the test lasted 30 s. In CE static balance condition, the participants stood upright with a bipedal stance (as described above) for 30 s with closed eyes. In OE+foam and CE+foam, the respective static OE and CE tests were performed with a balance foam pad (model LivePro 48 x 40 x 6 cm, Nantong Liveup Sports. CO. LTD, Nantong, China) placed under the participant’s feet.

The OE, CE, OE+foam, and CE+foam tests were performed also in dynamic conditions on the same platform used in tilting board modality. The platform was calibrated according to the manufacturer’s guidelines and tailored to the participants’ body mass to ensure an inclination of 5° on the three planes. The participants stood upright with the feet positioned in parallel maintaining a distance equal to that of the hips. The dynamic tests required to keep the centre of pressure (CoP) as close as possible to a permanent marker located in the centre of the screen. In static and dynamic tests, the area and perimeter described by the CoP sway was measured. Only in the static tests, the mean speed of the anteroposterior (AP) and mediolateral (ML) sway was calculated by the software provided by the manufacturer.

#### Muscle activation

Muscle activation levels were determined during MVC and balance tests by detecting the sEMG signal from the *vastus lateralis*, *biceps femoris*, *gastrocnemius medialis*, and *tibialis anterior* muscles. The skin area under the electrodes was shaved, cleaned with ethyl alcohol, abraded gently with fine sandpaper and prepared with a conductive cream (Nuprep®, Weaver and Co., Aurora, USA) to achieve an inter-electrode impedance below 2000 Ω. sEMG signal was detected by two Ag/AgCl rounded electrodes with solid hydrogel (mod H124SG Kendall ARBO; diameter: 10 mm; inter-electrodes distance: 20 mm; Kendall, Donau, Germany). Following the European Recommendations for Surface Electromyography [20], the electrodes were placed along the direction of the muscle fibres, between the tendon and the motor point. At the end of the first session, the electrode placement was marked on a transparent sheet, together with some skin landmarks (e.g., moles, scars, angiomas) for reliability purposes. The electrodes were equipped with a probe (probe mass: 8.5 g, BTS Inc., Milano, Italy) that permitted the detection and the transfer of the sEMG signal by wireless modality, acquired at 1000 Hz, amplified (gain: 2000, impedance and the common rejection mode ratio of the equipment are >1015 Ω//0.2 pF and 60/10 Hz 92 dB, respectively), and driven to a wireless EMG system (FREEEMG 300, BTS Inc., Milano, Italy) that digitised (1000 Hz) and filtered (filter type: IV-order Butterworth filter; bandwidth: 10–500 Hz) the raw signals.

The sEMG signals from both the MVC and BC tests were analysed in time-domain: a 250-ms mobile window was used for the computation of the signal root mean square (sEMG RMS). In MVC tests, the average of the RMS corresponding to the central 1 s was analysed. In BC tests, the sEMG RMS was averaged over the entire test duration (30 s). Thereafter, the sEMG RMS of the BC tests was normalised for the MVC sEMG RMS for each muscle.

### Passive stretching protocol

PS protocol consisted of four exercises involving the hip extensor/flexor muscles and the ankle dorsi/plantarflexor muscles of both limbs. Each exercise consisted of five 45-s elongations extended to the maximum point of perceived discomfort, alternating the limbs [17]. Stretching protocol had a total duration of 1800 s (450 s x 4 exercises). To ensure that the exercises were performed in passive modality, the sEMG signal was checked. During the exercises, the participants lay supine on a medical bed. For the hip flexor muscles, the participant was placed with the pelvis on the lower edge of the medical bed with the non-stretched limb resting on an additional bed placed in series with the first one. The operator passively flexed the knee while extending simultaneously the hip. For hip extensor muscles, the hip was passively flexed with the knee fully extended. For the plantarflexor muscles, the foot was placed just beyond the lower edge of the bed and passively dorsiflexed. For the dorsiflexor muscles, the foot was passively positioned in plantar flexion. In both ankle stretching exercises, the knee was maintained in full extension.

### Statistical analysis

Statistical analysis was performed using a statistical software package (IBM SPSS Statistics v. 26, Armonk, NY, USA). The Shapiro-Wilk’s test was used to check the normal distribution of the sampling. The baseline values of the two experimental sessions were utilised to calculate the inter-day reliability. To this purpose, the intraclass correlation coefficient (ICC) and the standard error of the measurement (SEM%) were calculated. The ICC was interpreted as follows: ≥0.90: *very high*; 0.89–0.70: *high*; 0.69–0.50: *moderate*. A two-way repeated measures analysis of covariance (ANCOVA) [time: 3 levels (POST, POST_15_, POST_30_); condition: 2 levels (STR, CTRL)] was used to check for differences between conditions over time, using the values in PRE as covariate. The ANCOVA effect size was evaluated with partial eta squared (*η*_*p*_^*2*^) and classified as follows: <0.06: *small*; if, 0.06–0.14: *medium*; and >0.14: *large* [21]. Multiple comparisons were perfomed applying the Bonferroni’s correction. The Cohen’s *d* ES was calculated and interpreted as follows: 0.00–0.19: *trivial*; 0.20–0.59: *small*; 0.60–1.19: *moderate*; 1.20–1.99: *large*; ≥2.00: *very large* (https://www.cem.org/effect-size-calculator). Statistical significance was set with *P*<0.05. If not otherwise stated data are presented in mean(SD).

## Results

### Reliability

All variables evaluated showed intersession reliability values ranging from *high* to *very high* (ICC range: 0.728–0.998). The SEM% values ranged from 0.9% to 8.9% (Table 1).

**Table 1:** Intersession repeatability of range of motion measurements (ROM), maximum voluntary contraction (MVC), root mean square of the surface electromyographic signal (sEMG RMS), area and perimeter of the center of pressure (CoP), velocity in the antero-posterior (AP) and mediolateral (ML) direction.

		CTRL PRE	STR PRE	ICC	SEM%
		m(SD)	m(SD)		
**ROM (°)**	Hip extension	27(4)	26(4)	0.974	2.5
	Hip flexion	81(15)	82(16)	0.994	1.5
	Ankle Plantarflexion	31(5)	30(5)	0.992	1.5
	Ankle Dorsiflexion	37(5)	37(4)	0.982	1.8
**MVC (N)**	knee extensors	595(125)	593(124)	0.997	1.1
	Knee flexors	433(81)	432(79)	0.997	1.0
	Plantar flexors	758(142)	751(147)	0.976	3.0
	Dorsiflexors	480(90)	479(88)	0.998	0.9
**EMG RMS (mV)**	*Vastus lateralis*	0.726(0.10)	0.728(0.10)	0.992	1.3
	*Biceps femoris*	0.567(0.08)	0.564(0.08)	0.991	1.3
	*Gastrocnemius medialis*	0.538(0.08)	0.534(0.07)	0.991	1.3
	*Tibialis anterior*	0.426(0.06)	0.424(0.06)	0.982	1.8
**Static test**	OE	352(77)	349(75)	0.987	2.5
	CE	467(103)	463(106)	0.984	2.8
**CoP area (cm** ^**2**^ **)**	OE+foam	439(91)	436(89)	0.981	2.8
	CE+foam	598(109)	596(111)	0.979	2.7
**Static test**	OE	387(85)	385(77)	0.984	2.7
	CE	486(113)	497(111)	0.978	3.4
**CoP perimeter (cm)**	OE+foam	461(96)	473(92)	0.982	2.7
	CE+foam	616(117)	605(113)	0.975	3.0
**Static test**	OE	5.8(1.2)	6.1(0.8)	0.728	8.9
	CE	6.4(1.3)	6.4(1.3)	0.934	5.1
**Speed AP (cm·s** ^**-1**^ **)**	OE+foam	5.8(0.9)	5.8(0.8)	0.842	5.8
	CE+foam	6.4(0.9)	6.2(0.8)	0.793	6.1
**Static test**	OE	3.7(0.5)	3.8(0.5)	0.845	5.3
	CE	4.0(0.6)	4.0(0.5)	0.921	3.7
**Speed ML (cm·s** ^**-1**^ **)**	OE+foam	3.7(0.5)	3.7(0.5)	0.900	4.1
	CE+foam	4.1(0.6)	4.1(0.6)	0.901	4.4
**Dynamic test**	OE	1156(251)	1164(248)	0.981	3.0
	CE	1541(340)	1533(352)	0.978	3.3
**CoP area (cm** ^**2**^ **)**	OE+foam	1448(301)	1440(294)	0.971	3.5
	CE+foam	1974(361)	1960(368)	0.979	2.7
**Dynamic test**	OE	1179(277)	1189(255)	0.976	3.5
**CoP perimeter (cm)**	CE	1664(347)	1652(358)	0.971	3.6
	OE+foam	1506(310)	1489(306)	0.980	2.9
	CE+foam	2112(375)	2128(379)	0.972	3.0

ICC: intraclass correlation coefficient, SEM%: percentage of the standard error of the measurement. OE: eyes open, CE: eyes closed, OE+foam: eyes open with foam, CE+foam: eyes closed with foam.

### ROM, MVC and muscle activation

The time-course of PS-induced changes in ROM for the hip and ankle joints, MVC of the knee extensor/flexor and ankle dorsi/plantarflexor muscles and sEMG RMS for the *vastus lateralis*, *biceps femoris*, *gastrocnemius medialis* and *tibialis anterior* muscles are shown in Table 2.

**Table 2:** Time course of range of motion (ROM), maximum voluntary contraction (MVC), and root mean square of the surface electromyographic signal (sEMG RMS) in the control (CTRL) and stretching session (STR).

		CTRL				STR					
		PRE	POST	POST_15_	POST_30_	PRE	POST	POST_15_	POST_30_	Time effect	Condition x time interaction
		m(SD)	m(SD)	m(SD)	m(SD)	m(SD)	m(SD)	m(SD)	m(SD)		
**ROM (°)**	Hip	26(4)	26(4)	27(4)	27(4)	26(4)	28(4)^a^^.^^b^	28(4)^a^	27(4)	*F* = 12.21	*F* = 20.64
										*P<0*.*001*	*P<0*.*001*
	extension										
										*η*_*p*_^*2*^ = 0.14	*η*_*p*_^*2*^ = 0.22
	Hip	81(15)	82(16)	81(16)	80(15)	82(16)	86(16)^a^^.^^b^	84(16)^a^	82(16)	*F* = 16.21	*F* = 13.58
										*P<0*.*001*	*P<0*.*001*
	flexion									*η*_*p*_^*2*^ = 0.40	*η*_*p*_^*2*^ = 0.36
	Ankle	31(5)	30(5)	31(5)	30(5)	30(5)	32(5)^a^^.^^b^	31(5)^a^	31(5)	*F* = 9.03	*F* = 18.54
										*P<0*.*001*	*P<0*.*001*
	Plantarflexion									*η*_*p*_^*2*^ = 0.11	*η*_*p*_^*2*^ = 0.20
	Ankle	37(5)	37(5)	37(5)	37(5)	37(4)	39(5) ^a^^.^^b^	38(5)^a^	37(5)	*F* = 13.42	*F* = 17.18
										*P<0*.*001*	*P<0*.*001*
	Dorsiflexion										
										*η*_*p*_^*2*^ = 0.15	*η*_*p*_^*2*^ = 0.19
**MVC (N)**	Knee extensors	595(125)	593(123)	591(124)	593(122)	593(124)	528(127)^a^^.^^b^	569(128)^a^^.^^b^	590(125)	*F* = 0.86	*F* = 16.08
										*P* = 0.668	*P<0*.*001*
										*η*_*p*_^*2*^ = 0.006	*η*_*p*_^*2*^ = 0.39
	Knee flexors	433(81)	432(79)	431(81)	431(80)	432(79)	399(79)^a^^.^^b^	419(81)^a^^.^^b^	430(79)	*F* = 1.45	*F* = 18.66
										*P* = 0.24	*P<0*.*001*
										*η*_*p*_^*2*^ = 0.02	*η*_*p*_^*2*^ = 0.35
	Plantarflexors	758(142)	757(148)	754(144)	753(147)	751(147)	690(155)^a^^.^^b^	717(148)^a^	749(146)	*F* = 21.38	*F* = 23.22
										*P<0*.*001*	*P<0*.*001*
										*η*_*p*_^*2*^ = 0.22	*η*_*p*_^*2*^ = 0.24
	Dorsiflexors	480(90)	474(88)	480(90)	479(92)	479(88)	443(83) ^a^^.^^b^	467(87) ^a^	479(90)	*F* = 16.90	*F* = 10.90
										*P<0*.*001*	*P<0*.*001*
										*η*_*p*_^*2*^ = 0.33	*η*_*p*_^*2*^ = 0.22
**EMG RMS (mV)**	*Vastus lateralis*	0.73(.10)	0.72(.10)	0.72(.11)	0.73(.11)	0.73(.10)	0.67(.11)^a^^.^^b^	0.70(.11)^a^	0.73(.11)	*F* = 17.90	*F* = 19.15
										*P<0*.*001*	*P<0*.*001*
										*η*_*p*_^*2*^ = 0.27	*η*_*p*_^*2*^ = 0.21
	*Biceps femoris*	0.57(.08)	0.57(.08)	0.56(.08)	0.56(.08)	0.56(.08)	0.52(.09)^a^^.^^b^	0.55(.08)^a^	0.56(.08)	*F* = 11.50	*F* = 11.34
										*P<0*.*001*	*P<0*.*001*
										*η*_*p*_^*2*^ = 0.23	*η*_*p*_^*2*^ = 0.22
	*Gastrocnemius medialis*	0.54(.08)	0.54(.08)	0.53(.07)	0.53(.08)	0.53(.07)	0.49(.08)^a^^.^^b^	0.51(.08)^a^	0.54(.07)	*F* = 11.79	*F* = 15.82
										*P<0*.*001*	*P<0*.*001*
										*η*_*p*_^*2*^ = 0.23	*η*_*p*_^*2*^ = 0.26
	*Tibialis anterior*	0.43(.06)	0.42(.06)	0.42(.06)	0.43(.06)	0.42(.06)	0.39(.05)^a^^.^^b^	0.41(.05)^a^	0.42(.06)	*F* = 15.19	*F* = 6.10
										*P<0*.*001*	*P<0*.*001*
										*η*_*p*_^*2*^ = 0.32	*η*_*p*_^*2*^ = 0.18

^a^: p<0.05 vs PRE

^b^: p<0.05 vs CTRL.

The ANCOVA found a condition x time interaction and a main effect for time in all ROM measurements. Hip and ankle ROM increased in STR at POST and POST_15_ in all tests (Δ%-range: 6.1%– 6.8% and 3.1%– 4.0%; *d-*range: 0.41–0.44 and 0.22–0.44; POST and POST_15_, respectively, *P*<0.001 in all comparisons). There was no change in ROM in CTRL. The ANCOVA disclosed an increase in all ROM measurements in STR compared to CTRL at POST (Δ%-range: 4.9%– 6.8%; *d-*range: 0.40–0.44, *P*<0.001 in all comparisons).

The ANCOVA found a condition x time interaction in all MVC measurements and a main effect for *time* in MVC of the dorsi/plantarflexor muscles. Regardless of the muscle action, MVC decreased in STR at POST and POST_15_ (Δ%-range: 7.4%– 11.0% and 2.4%– 4.0%; *d-*range: -0.51 –-0.40 and -0.23 –-0.19; POST and POST_15_, respectively, *P*<0.001 in all comparisons). There was no change in MVC in CTRL. A between-condition difference was found at POST for all MVCs (Δ%-range of decrease: 7.7%– 11.1%; *d*-range: -0.24 –-0.19; *P*-range: 0.003 –<0.001).

The ANCOVA revealed a condition x time interaction and a main effect for time in the sEMG RMS values in all evaluated muscles. sEMG RMS decreased in STR at POST and POST_15_ in all muscles (Δ%-range: 7.3%– 7.9% and 2.3%– 4.0%; *d*-range: -0.56 –-0.46 and -0.28 –-0.18; POST and POST_15_, respectively, *P*-range: 0.001 –<0.001). No changes in sEMG RMS were found in CTRL. A between-condition difference was found in POST (Δ%-range of decrease: 6.7%– 7.6%; *d*-range: -0.62 –-0.47; *P*-range: 0.003 –<0.001).

### Static and dynamic balance tests

No condition x time interaction (*P*>0.05) or main effect for *time* (*P*>0.05) were retrieved for the CoP area (Table 3) and perimeter (Table 4) neither in static nor in dynamic tests. No between-condition differences were found at any time point.

**Table 3:** Time course of centre of pressure (CoP) area in the control (CTRL) and stretching session (STR) during the static and dynamic tests.

		CTRL				STR					
	CoP area (mm^2^)	PRE	POST	POST_15_	POST_30_	PRE	POST	POST_15_	POST_30_	Time effect	Condition x time interaction
		m(SD)	m(SD)	m(SD)	m(SD)	m(SD)	m(SD)	m(SD)	m(SD)		
**Static tests**	OE	352(77)	349(79)	349(74)	350(75)	349(75)	353(80)	350(77)	351(76)	*F* = 0.40	*F* = 2.36
										*P* = 0.75	*P* = 0.08
										*η*_*p*_^*2*^ = 0.02	*η*_*p*_^*2*^ = 0.09
	CE	467(103)	468(109)	468(107)	468(111)	463(106)	470(110)	467(107)	467(106)	*F* = 0.60	*F* = 0.68
										*P* = 0.62	*P* = 0.59
										*η*_*p*_^*2*^ = 0.03	*η*_*p*_^*2*^ = 0.03
	OE+foam	439(91)	434(89)	432(87)	441(99)	436(89)	441(91)	438(89)	443(94)	*F* = 2.85	*F* = 0.96
										*P* = 0.07	*P* = 0.41
										*η*_*p*_^*2*^ = 0.09	*η*_*p*_^*2*^ = 0.04
	CE+foam	598(109)	600(122)	590(109)	600(122)	596(111)	596(110)	598(110)	599(112)	*F* = 0.41	*F* = 1.05
										*P* = 0.75	*P* = 0.38
										*η*_*p*_^*2*^ = 0.02	*η*_*p*_^*2*^ = 0.04
**Dynamic tests**	OE	1156(251)	1151(262)	1151(244)	1154(247)	1164(248)	1165(223)	1143(233)	1153(248)	*F* = 1.93	*F* = 0.84
										*P* = 0.13	*P* = 0.48
										*η*_*p*_^*2*^ = 0.03	*η*_*p*_^*2*^ = 0.10
	CE	1541(340)	1543(359)	1546(353)	1546(366)	1533(352)	1548(370)	1532(359)	1538(351)	*F* = 0.34	*F* = 0.49
										*P* = 0.80	*P* = 0.69
										*η*_*p*_^*2*^ = 0.005	*η*_*p*_^*2*^ = 0.007
	OE+foam	1448(301)	1434(294)	1425(287)	1455(325)	1440(294)	1461(289)	1422(281)	1446(301)	*F* = 3.21	*F* = 1.09
										*P* = 0.20	*P* = 0.35
										*η*_*p*_^*2*^ = 0.04	*η*_*p*_^*2*^ = 0.02
	CE+foam	1974(361)	1981(402)	1947(360)	1979(401)	1960(368)	1964(412)	1976(394)	1971(384)	*F* = 0.55	*F* = 0.77
										*P* = 0.65	*P* = 0.51
										*η*_*p*_^*2*^ = 0.007	*η*_*p*_^*2*^ = 0.01

OE: eyes open, CE: eyes closed, OE+foam: eyes open with foam, CE+foam: eyes closed with foam.

**Table 4:** Time course of centre of pressure (CoP) perimeter in the control (CTRL) and stretching session (STR) during the static and dynamic tests.

		CTRL				STR					
	CoP perimeter (mm)	PRE	POST	POST_15_	POST_30_	PRE	POST	POST_15_	POST_30_	Time effect	Condition x time interaction
		m(SD)	m(SD)	m(SD)	m(SD)	m(SD)	m(SD)	m(SD)	m(SD)		
**Static tests**	OE	387(85)	384(82)	366(78)	375(77)	385(77)	360(86)	361(80)	365(81)	*F* = 0.44	*F* = 2.31
										*P* = 0.62	*P* = 0.09
										*η*_*p*_^*2*^ = 0.02	*η*_*p*_^*2*^ = 0.08
	CE	486(113)	491(114)	491(113)	482(114)	497(111)	498(119)	495(111)	486(114)	*F* = 0.63	*F* = 0.61
										*P* = 0.51	*P* = 0.43
										*η*_*p*_^*2*^ = 0.02	*η*_*p*_^*2*^ = 0.03
	OE+foam	461(96)	460(93)	459(91)	472(103)	473(92)	463(97)	464(93)	474(100)	*F* = 1.95	*F* = 0.99
										*P* = 0.06	*P* = 0.55
										*η*_*p*_^*2*^ = 0.08	*η*_*p*_^*2*^ = 0.02
	CE+foam	616(117)	618(126)	614(117)	618(126)	605(113)	608(117)	622(113)	617(120)	*F* = 0.39	*F* = 1.01
										*P* = 0.81	*P* = 0.32
										*η*_*p*_^*2*^ = 0.01	*η*_*p*_^*2*^ = 0.04
**Dynamic tests**	OE	1179(277)	1209(272)	1186(259)	1177(254)	1189(255)	1188(239)	1189(242)	1188(268)	*F* = 1.81	*F* = 0.83
										*P* = 0.21	*P* = 0.51
										*η*_*p*_^*2*^ = 0.02	*η*_*p*_^*2*^ = 0.09
	CE	1664(347)	1666(381)	1654(371)	1639(373)	1652(358)	1672(400)	1624(370)	1646(376)	*F* = 0.44	*F* = 0.42
										*P* = 0.62	*P* = 0.64
										*η*_*p*_^*2*^ = 0.01	*η*_*p*_^*2*^ = 0.01
	OE+foam	1506(310)	1491(312)	1496(304)	1499(338)	1489(306)	1505(308)	1450(287)	1518(325)	*F* = 1.91	*F* = 1.07
										*P* = 0.07	*P* = 0.41
										*η*_*p*_^*2*^ = 0.01	*η*_*p*_^*2*^ = 0.02
	CE+foam	2112(375)	2139(418)	2064(385)	2118(413)	2128(379)	2101(441)	2134(414)	2109(411)	*F* = 0.51	*F* = 0.71
										*P* = 0.72	*P* = 0.78
										*η*_*p*_^*2*^ = 0.01	*η*_*p*_^*2*^ = 0.01

OE: eyes open, CE: eyes closed, OE+foam: eyes open with foam, CE+foam: eyes closed with foam.

The time-course of PS-induced changes in AP and ML speed during the static tests are shown in Table 5. With the exception of the OE condition, the ANCOVA revealed a condition x time interaction. Furthermore, a main effect for time was found in the AP speed in OE and OE+foam and in the ML speed in CE+foam. After stretching, the AP speed decreased in STR at POST (Δ%-range: 5.0%– 10.2%; *d*-range:-0.62 –-0.37, *P*<0.001 in all comparisons), POST_15_ (Δ%-range: 3.0%– 7.0%; *d*-range: -0.37 –-0.25, *P*<0.001 in all comparisons) and only for the CE+foam condition at POST_30_ (Δ% = 4.2%; *d* = -0.35, *P* = 0.01). No changes were found in CTRL. Between-condition differences were found at POST (Δ%-range: 6.0%– 7.7%; *d*-range: -1.25 –-0.59, *P*<0.001 in all comparisons), and in CE and OE+foam conditions also in POST_15_ (Δ% = 3.1% and 4.2%; *d*: -0.69 and -0.25; in CE and OE+foam respectively, *P*<0.001 in all comparisons). Similarly, after stretching the ML speed decreased in STR at POST (Δ%-range: 8.4%– 18.7%; *d*-range: -1.29 –-0.55, *P*<0.001 in all comparisons), POST_15_ (Δ%-range: 5.0%– 15.0%; *d*-range: -1.08–0.40, *P*<0.001 in all comparisons), and, except for the OE+foam, at POST_30_ (Δ%-range: 2.5%– 10.9%; *d*-range: -0.72 –-0.40, *P*-range: 0.01–0.006). No changes were found in CTRL. Between-condition differences were found at POST (Δ%-range of decrease: 9.0%– 18.8%; *d*-range:-1.14 –-0.59, *P*<0.001 in all comparisons), POST_15_ (Δ%-range of decrease: 4.6%– 15.3%; *d*-range:-0.99 –-0.36, *P*<0.001 in all comparisons), and, with the exception of the OE+foam, at POST_30_ (Δ%-range of decrease: 3.2%– 10.9%; *d*-range:-0.79 –-0.20, *P*-range: 0.01–0.001).

**Table 5:** Time course of anteroposterior (AP) and mediolateral (ML) sway speed in the control (CTRL) and stretching session (STR) during the static tests.

		CTRL				STR					
	Speed AP	PRE	POST	POST_15_	POST_30_	PRE	POST	POST_15_	POST_30_	Time effect	Condition x time interaction
	(cm·s^-1^)	m(SD)	m(SD)	m(SD)	m(SD)	m(SD)	m(SD)	m(SD)	m(SD)		
**Static tests**	OE	5.8(1.9)	5.8(0.9)	5.8(0.9)	5.9(0.9)	6.1(0.8)	5.6(0.9)^a^^.^^b^	5.9(0.8)	5.9(0.8)	*F* = 14.9	*F* = 1.9
										*P<0*.*001*	*P* = 0.12
										*η*_*p*_^*2*^ = 0.17	*η*_*p*_^*2*^ = 0.03
	CE	6.4(1.3)	6.4(1.3)	6.3(1.4)	6.3(1.3)	5.8(0.8)	5.4(0.8)^a^^.^^b^	5.5(0.8)^a^^.^^b^	5.8(0.8)	*F* = 0.91	*F* = 10.12
										*P* = 0.44	*P<0*.*001*
										*η*_*p*_^*2*^ = 0.01	*η*_*p*_^*2*^ = 0.12
	OE+foam	5.81(0.9)	5.9(0.8)	5.7(0.8)	5.8(0.9)	5.8(0.8)	5.5(0.8)^a^^.^^b^	5.5(0.8)^a^^.^^b^	5.8(0.8)	*F* = 4.04	*F* = 7.94
										*P* = 0.005	*P<0*.*001*
										*η*_*p*_^*2*^ = 0.06	*η*_*p*_^*2*^ = 0.10
	CE+foam	6.4(0.9)	6.2(1.0)	6.1(0.9)	6.2(0.9)	6.2(0.8)	5.7(0.8)^a^^.^^b^	5.9(0.9)^a^	5.9(0.8)^a^	*F* = 0.34	*F* = 3.98
										*P* = 0.79	*P* = 0.009
										*η*_*p*_^*2*^ = 0.005	*η*_*p*_^*2*^ = 0.05
	**Speed ML**	**PRE**	**POST**	**POST**_**15**_	**POST**_**30**_	**PRE**	**POST**	**POST**_**15**_	**POST**_**30**_	Time effect	Condition x time interaction
	**(cm·s** ^**-1**^ **)**	**m(SD)**	**m(SD)**	**m(SD)**	**m(SD)**	**m(SD)**	**m(SD)**	**m(SD)**	**m(SD)**		
**Static tests**	OE	3.7(0.5)	3.8(0.5)	3.9(0.6)	3.8(0.5)	3.8(0.5)	3.4(0.5)^a^^.^^b^	3.5(0.5)^a^^.^^b^	3.6(0.5)^a^^.^^b^	*F* = 1.36	*F* = 18.68
										*P* = 0.26	*P<0*.*001*
										*η*_*p*_^*2*^ = 0.02	*η*_*p*_^*2*^ = 0.20
	CE	4.0(0.6)	4.0(0.6)	4.0(0.6)	4.0(0.5)	4.0(0.5)	3.6(0.4)^a^^.^^b^	3.8(0.5)^a^^.^^b^	3.9(0.5)^a^^.^^b^	*F* = 2.27	*F* = 16.56
										*P* = 0.08	*P<0*.*001*
										*η*_*p*_^*2*^ = 0.03	*η*_*p*_^*2*^ = 0.19
	OE+foam	3.7(0.5)	3.7(0.5)	3.7(0.6)	3.7(0.5)	3.7(0.5)	3.4(0.5)^a^^.^^b^	3.5(0.5)^a^^.^^b^	3.7(0.5)	*F* = 0.24	*F* = 14.51
										*P* = 0.87	*P<0*.*001*
										*η*_*p*_^*2*^ = 0.003	*η*_*p*_^*2*^ = 0.17
	CE+foam	4.1(0.6)	4.1(0.7)	4.1(0.7)	4.1(0.5)	4.1(0.6)	3.4(0.5)^a^^.^^b^	3.5(0.5)^a^^.^^b^	3.7(0.5)^a^^.^^b^	*F* = 17.58	*F* = 22.38
										*P<0*.*001*	*P<0*.*001*
										*η*_*p*_^*2*^ = 0.19	*η*_*p*_^*2*^ = 0.24

OE: eyes open, CE: eyes closed, OE+foam: eyes open with foam, CE+foam: eyes closed with foam.

^a^: p<0.05 vs PRE

^b^: p<0.05 STR vs CTRL.

The ANCOVA reported condition x time interactions for activation values in static tests, apart from the *gastrocnemius medialis* in CE, *biceps femoris* in OE+foam and CE+foam, *vastus lateralis*, and *tibialis anterior* in CE+foam. A main effect for time was also reported in all muscles except for the *gastrocnemius medialis* in CE+foam (Table 6). Muscle activation increased in STR in all muscles, in all tests at POST (Δ%-range: 4.6%– 11.7%; *d*-range: 0.20–0.90, *P*<0.001 in all comparisons) and, with some exceptions, at POST_15_ (Δ%-range of increases: 3.5%– 7.2%; *d*-range: 0.15–0.72, *P*-range: 0.005 –<0.001). No differences were found in CTRL. Between-condition differences were found in POST (Δ%-range of increase: 4.7%– 23.0%; *d*-range: 0.15–0.97, *P* range: 0.02 –<0.001) and, with some exceptions, at POST_15_ (Δ%-range of increase: 4.8%– 18.6%; *d*-range: 0.25–0.82, *P*-range: 0.03 –<0.001).

**Table 6:** Percentage of muscle activation time course in the control (CTRL) and stretching session (STR) during the static tests.

Muscle activation level (% sEMG RMS during MVC at Pre)												
			CTRL				STR					
			PRE	POST	POST_15_	POST_30_	PRE	POST	POST_15_	POST_30_	Time effect	Condition x time interaction
			m(SD)	m(SD)	m(SD)	m(SD)	m(SD)	m(SD)	m(SD)	m(SD)		
**Static tests**	**OE**	VL	2.5(0.8)	2.4(0.7)	2.5(0.4)	2.4(0.6)	2.6(0.5)	2.8(0.5)^a^^.^^b^	2.7(0.5)^b^	2.6(0.5)	*F* = 16.16	*F* = 6.91
											*P*<0.001	*P* = 0.001
											*η*_*p*_^*2*^ = 0.18	*η*_*p*_^*2*^ = 0.09
		BF	2.4(0.6)	2.4(0.6)	2.3(0.7)	2.5(0.6)	2.5(0.4)	2.8(0.4)^a^^.^^b^	2.7(0.4)^a^^.^^b^	2.6(0.4)	*F* = 7.77	*F* = 7.31
											*P<0*.*001*	*P<0*.*001*
											*η*_*p*_^*2*^ = 0.10	*η*_*p*_^*2*^ = 0.09
		GM	5.5(1.3)	5.2(1.2)	5.0(1.0)	5.0(1.1)	5.4(1.2)	5.8(1.2)^a^^.^^b^	5.6(1.1)^b^	5.3(1.1)^b^	*F* = 6.60	*F* = 4.56
											*P<0*.*001*	*P* = 0.04
											*η*_*p*_^*2*^ = 0.17	*η*_*p*_^*2*^ = 0.06
		TA	4.5(0.7)	4.4(0.8)	4.5(0.8)	4.5(0.6)	4.7(0.7)	5.0(0.8)^a^^.^^b^	4.9(0.9)	4.7(0.7)	*F* = 2.76	*F* = 3.68
											*P* = 0.04	*P* = 0.01
											*η*_*p*_^*2*^ = 0.04	*η*_*p*_^*2*^ = 0.05
	**CE**	VL	2.6(0.8)	2.7(0.8)	2.7(0.8)	2.8(0.7)	2.7(0.5)	3.2(0.6)^a^^.^^b^	3.1(0.6) ^a^^.^^b^	2.9(0.6)	*F* = 10.23	*F* = 3.69
											*P<0*.*001*	*P* = 0.01
											*η*_*p*_^*2*^ = 0.12	*η*_*p*_^*2*^ = 0.05
		BF	2.7(0.**7**)	2.6(0.8)	2.6(0.7)	2.5(0.8)	2.8(0.6)	3.1(0.6)^a^^.^^b^	3.0(0.6)^a^^.^^b^	2.9(0.7)	*F* = 5.74	*F* = 3.08
											*P = 0*.*001*	*P* = 0.03
											*η*_*p*_^*2*^ = 0.07	*η*_*p*_^*2*^ = 0.04
		GM	5.2(0.7)	5.2(0.8)	5.1(0.7)	5.4(0.7)	5.4(0.8)	5.9(0.9)^a^^.^^b^	5.7(0.9)^b^	5.5(0.8)	*F* = 2.86	*F* = 2.52
											*P* = 0.04	*P* = 0.06
											*η*_*p*_^*2*^ = 0.04	*η*_*p*_^*2*^ = 0.03
		TA	5.3(1.2)	5.4(1.3)	5.2(1.1)	5.6(1.0)	5.3(2.0)	5.8(1.2)^a^^.^^b^	5.6(1.0)^a^^.^^b^	5.3(1.0)	*F* = 3.54	*F* = 8.60
											*P* = 0.02	*P<0*.*001*
											*η*_*p*_^*2*^ = 0.05	*η*_*p*_^*2*^ = 0.11
	**OE+foam**	VL	2.4(0.7)	2.4(0.6)	2.4(0.7)	2.4(0.7)	2.6(0.4)	2.9(0.4)^a^^.^^b^	2.9(0.5) ^a^^.^^b^	2.8(0.5)	*F* = 8.76	*F* = 2.6
											*P<*0.001	*P* = 0.05
											*η*_*p*_^*2*^ = 0.11	*η*_*p*_^*2*^ = 0.04
		BF	2.5(0.6)	2.4(0.6)	2.4(0.8)	2.6(0.7)	2.6(0.5)	2.7(0.5)^b^	2.6(0.7)	2.6(0.6)	*F* = 18.51	*F* = 1.88
											*P<0*.*001*	*P* = 0.13
											*η*_*p*_^*2*^ = 0.20	*η*_*p*_^*2*^ = 0.03
		GM	5.1(1.1)	5.0(1.1)	5.2(1.1)	5.2(1.2)	5.3(1.1)	5.8(1.2)^a^^.^^b^	5.6(1.2)^a^^.^^b^	5.3(1.1)	*F* = 3.52	*F* = 5.68
											*P* = 0.02	*P* = 0.001
											*η*_*p*_^*2*^ = 0.05	*η*_*p*_^*2*^ = 0.07
		TA	4.4(0.7)	4.2(0.8)	4.2(1.0)	4.5(0.9)	4.3(0.8)	4.6(0.8)^b^	4.5(0.9)	4.4(0.8)	*F* = 12.09	*F* = 4.19
											*P<0*.*001*	*P* = 0.007
											*η*_*p*_^*2*^ = 0.14	*η*_*p*_^*2*^ = 0.05
	**CE+foam**	VL	3.2(0.6)	3.1(0.9)	3.0(0.8)	3.1(0.6)	2.8(0.5)	3.0(0.5)^a^^.^^b^	2.9(0.4)	2.9(0.5)	*F* = 24.00	*F* = 2.00
											*P<*0.001	*P* = 0.11
											*η*_*p*_^*2*^ = 0.25	*η*_*p*_^*2*^ = 0.03
		BF	5.8(1.4)	5.7(1.3)	5.4(1.2)	5.8(1.3)	6.2(1.1)	6.6(1.2)^b^	6.4(1.2)	6.2(1.1)	*F* = 18.51	*F* = 1.88
											*P<0*.*001*	*P* = 0.13
											*η*_*p*_^*2*^ = 0.20	*η*_*p*_^*2*^ = 0.03
		GM	5.8(0.9)	5.8(1.0)	5.7(1.1)	5.6(1.1)	6.0(1.0)	6.4(1.0)^a^^.^^b^	6.2(1.1)^b^	6.1(1.0)	*F* = 1.91	*F* = 4.79
											*P* = 0.12	*P* = 0.003
											*η*_*p*_^*2*^ = 0.03	*η*_*p*_^*2*^ = 0.06
		TA	5.2(1.3)	5.2(1.4)	5(1.1)	5.1(1.5)	5.1(1.3)	5.4(1.2) ^a^^.^^b^	5.3(1.3)	5.2(1.2)	*F* = 4.44	*F* = 1.51
											*P* = 0.005	*P* = 0.21
											*η*_*p*_^*2*^ = 0.06	*η*_*p*_^*2*^ = 0.02

OE: eyes open, CE: eyes closed, OE+foam: eyes open with foam, CE+foam: eyes closed with foam. VL: *vastus lateralis*, BF: *biceps femoris*, GM: *gastrocnemius medialis*, TA: *tibialis anterior*.

^a^: p<0.05 vs PRE

^b^: p<0.05 STR vs CTRL.

The ANCOVA reported condition x time interactions in all muscles and in all conditions, apart from the activation in *vastus lateralis* in CE and *biceps femoris* in OE. A main effect for *time* was found in all conditions except fort *biceps femoris* in CE and CE+foam, in the *gastrocnemius medialis* in OE and CE+foam, and in the *tibialis anterior* muscle in all conditions (Table 7). Muscle activation increased in STR in all muscles and in all conditions, except for the *gastrocnemius medialis* and *tibialis anterior* muscles in CE+foam at POST (Δ%-range: 4.3%– 11.0%; *d*-range: 0.10–0.57, *P*-range: 0.005 –<0.001) and, with some exceptions, at POST_15_ (Δ%-range: 4.4%– 8.5%; *d*-range: 0.13–0.33, *P*-range: 0.04 –<0.001). No differences were found in CTRL. Between-condition differences were found at POST (Δ%-range of increases: 3.6%– 20.2%; *d*-range: 0.16–0.79, *P* range: 0.002–0.001) and only in some muscles and during some tests at POST_15_ (Δ%-range of increases: 2.5%– 11.6%; *d*-range: 0.12–0.54, *P*-range: 0.04 –<0.001).

**Table 7:** Time course of percentage muscle activation in the control (CTRL) and stretching session (STR) during the dynamic tests.

Muscle activation level (% sEMG RMS during MVC at Pre)												
			CTRL				STR					
			PRE	POST	POST_15_	POST_30_	PRE	POST	POST_15_	POST_30_	Time effect	Condition x time interaction
			m(SD)	m(SD)	m(SD)	m(SD)	m(SD)	m(SD)	m(SD)	m(SD)		
**Dynamic tests**	**OE**	VL	3.1(0.5)	2.9(0.8)	3.0(0.5)	3.0(0.8)	3.1(0.7)	3.5(0.7)^a^^.^^b^	3.3(0.6)	3.3(0.7)	*F* = 31.83	*F* = 4.34
											*P<0*.*001*	*P* = 0.005
											*η*_*p*_^*2*^ = 0.30	*η*_*p*_^*2*^ = 0.06
		BF	3.9(0.8)	4.1(0.7)	4.0(0.8)	4.0(0.7)	4.0(0.7)	4.3(0.7)^a^^.^^b^	4.1(0.8)^a^^.^^b^	4.1(0.8)	*F* = 5.56	*F* = 1.90
											*P* = 0.001	*P* = 0.13
											*η*_*p*_^*2*^ = 0.07	*η*_*p*_^*2*^ = 0.02
		GM	9.4(2.4)	9.4(2.5)	9.9(2.9)	9.6(2.3)	9.5(2.3)	10.5(2.6)^a^^.^^b^	10.3(2.5)^a^	10.1(2.5)^a^	*F* = 0.59	*F* = 3.50
											*P* = 0.624	*P* = 0.01
											*η*_*p*_^*2*^ = 0.008	*η*_*p*_^*2*^ = 0.05
		TA	8.5(1.7)	8.6(1.7)	8.6(2.2)	8.5(1.7)	8.6(1.7)	9.5(1.9)^a^^.^^b^	9.0(1.8)^a^	8.6(1.8)	*F* = 2.29	*F* = 6.73
											*P* = 0.08	*P<0*.*001*
											*η*_*p*_^*2*^ = 0.03	*η*_*p*_^*2*^ = 0.08
	**CE**	VL	3.0(1.1)	3.1(1.0)	3.0(1.0)	3.0(0.9)	3.1(1.0)	3.2(1.0) ^a^^.^^b^	3.2(1.1)	3.2(1.0)	*F* = 5.03	*F* = 1.40
											*P* = 0.002	*P = 0*.*25*
											*η*_*p*_^*2*^ = 0.06	*η*_*p*_^*2*^ = 0.02
		BF	4.6(1.1)	4.7(1.2)	4.6(1.3)	4.6(1.3)	4.6(1.2)	4.9(1.3)^a^^.^^b^	4.8(1.2)^b^	4.7(1.2)	*F* = 1.09	*F* = 5.61
											*P* = 0.36	*P* = 0.02
											*η*_*p*_^*2*^ = 0.02	*η*_*p*_^*2*^ = 0.07
		GM	10.4(2.8)	10.4(2.6)	10.1(2.3)	10.5(2.5)	10.4(2.4)	11.5(2.9)^a^^.^^b^	11.0(2.6)^a^^.^^b^	10.7(2.6)^a^	*F* = 2.26	*F* = 12.28
											*P* = 0.09	*P<0*.*001*
											*η*_*p*_^*2*^ = 0.03	*η*_*p*_^*2*^ = 0.14
		TA	8.8(1.9)	8.8(1.9)	8.9(1.9)	8.9(1.7)	8.9(1.7)	9.6(2.0)^a^^.^^b^	9.4(1.9)^a^^.^^b^	8.9(1.7)	*F* = 2.04	*F* = 6.19
											*P* = 0.11	*P<0*.*001*
											*η*_*p*_^*2*^ = 0.03	*η*_*p*_^*2*^ = 0.08
	**OE+foam**	VL	2.8(0.5)	2.8(0.6)	2.7(0.5)	2.8(0.6)	2.8(0.4)	2.9(0.5)^a^^.^^b^	2.9(0.4)	2.9(0.5)	*F* = 5.98	*F* = 2.00
											*P* = 0.001	*P* = 0.14
											*η*_*p*_^*2*^ = 0.08	*η*_*p*_^*2*^ = 0.03
		BF	4.2(1.0)	4.3(1.0)	4.1(0.8)	4.2(0.9)	4.2(0.8)	4.6(1.0)^a^^.^^b^	4.4(0.9)^a^^.^^b^	4.3(0.9)	*F* = 3.41	*F* = 3.12
											*P* = 0.02	*P* = 0.03
											*η*_*p*_^*2*^ = 0.05	*η*_*p*_^*2*^ = 0.04
		GM	9.4(2.3)	9.4(1.9)	9.4(2.4)	9.4(2.1)	9.4(2.0)	10.3(2.3)^a^^.^^b^	10.0(2.2)^a^^.^^b^	9.5(2.1)	*F* = 3.52	*F* = 5.68
											*P* = 0.02	*P* = 0.001
											*η*_*p*_^*2*^ = 0.05	*η*_*p*_^*2*^ = 0.07
		TA	8.3(1.8)	8.5(2.0)	8.4(1.7)	8.5(1.7)	8.4(1.7)	9.0(1.7)^a^^.^^b^	8.8(1.9)^a^	8.5(1.7)	*F* = 2.24	*F* = 5.38
											*P* = 0.09	*P* = 0.001
											*η*_*p*_^*2*^ = 0.03	*η*_*p*_^*2*^ = 0.07
	**CE+foam**	VL	2.9(0.7)	2.8(0.6)	2.7(0.5)	2.8(0.6)	2.8(0.7)	3.0(0.5)^a^^.^^b^	2.9(0.4)^a^^.^^b^	3.1(0.6)^a^^.^^b^	*F* = 9.06	*F* = 11.54
											*P<0*.*001*	*P<0*.*001*
											*η*_*p*_^*2*^ = 0.11	*η*_*p*_^*2*^ = 0.14
		BF	4.7(0.8)	4.6(0.4)	4.7(1.0)	4.6(0.9)	4.7(0.9)	5.0(0.9)^a^^.^^b^	4.8(0.3)	4.7(0.8)	*F* = 1.54	*F* = 4.36
											*P* = 0.20	*P* = 0.005
											*η*_*p*_^*2*^ = 0.02	*η*_*p*_^*2*^ = 0.06
		GM	9.5(2.2)	9.8(2.6)	9.7(2.2)	9.7(2.0)	9.7(2.1)	10.6(2.3)	10.2(2.4)	9.8(2.1)	*F* = 2.54	*F* = 3.64
											*P* = 0.06	*P* = 0.01
											*η*_*p*_^*2*^ = 0.03	*η*_*p*_^*2*^ = 0.05
		TA	10.2(1.7)	10.0(2.1)	10.3(2.6)	10.1(1.7)	10.1(1.9)	10.9(2.2)	10.6(2.0)	10.2(2.0)	*F* = 1.22	*F* = 5.34
											*P* = 0.30	*P* = 0.001
											*η*_*p*_^*2*^ = 0.02	*η*_*p*_^*2*^ = 0.07

OE: eyes open, CE: eyes closed, OE+foam: eyes open with foam, CE+foam: eyes closed with foam. VL: *vastus lateralis*, BF: *biceps femoris*, GM: *gastrocnemius medialis*, TA: *tibialis anterior*.

^a^: p<0.05 vs PRE

^b^: p<0.05 STR vs CTRL.

## Discussion

The present study aimed to evaluate the acute effects of a PS session on BC under both static and dynamic condition to highlight possible compensation mechanisms adopted by the vestibular and visual systems in response to an alteration of the afferent somatosensory feedback. Contrary to our hypothesis, the present results highlighted that following PS, static and dynamic BC was unchanged. However, greater activation of the lower limb muscles occurred to maintain the same level of BC under both conditions. Additionally, a reduced speed of CoP sway in the AP and ML directions occurred, indicating a possible deterioration in the BC. Lastly, it seems that no compensative mechanism from the vestibular and/or visual systems took place, as suggested by the observed levels of muscle activation during the tests with a limited somatosensory feedback (foam under the feet). Altogether, the current findings showed that PS did not alter the gross BC, albeit decreased the muscle efficiency involved in the BC maintenance.

### Preliminary considerations

ROM, MVC and muscle activation were measured to check the effectiveness of the adopted PS protocol. As expected, ROM increased after stretching while MVC and maximum muscle activation decreased. These effects persisted up to 15 min, returning to pre-stretch values within 30 min. In line with previous literature, these effects may be likely ascribed to the concomitant action of neuromuscular [i.e., alteration in somatosensory afferent feedback by type-I*a*, type-II (muscle spindles) [22], type-III (mechanoreceptors), and type-IV (metabo-/nociceptors) fibres [23, 24]] and mechanical mechanisms, including a decrease in muscle-tendon unit stiffness [6, 7, 25]. Importantly, previous studies argued that repeating balance tests can introduce a learning effect, thus biasing the possible PS-induced adaptation in BC [10]. In this study, the familiarisation period continued until the test results plateaued, in the attempt to account for the possible above-mentioned learning effect. Therefore, the high reliability values achieved in the present investigation can be explained also by this familiarisation process (Table 1). Noteworthy, given the present familiarization process was not done previously in all studies, it should be noted that the present results may not suffer from any possible learning effect, thus smoothing the findings. This could account for possible differences with previous studies.

### Balance tests

PS did not change gross BC under static condition, as witnessed by the similar CoP sway perimeter and area values observed among the different tests after stretching. However, greater activation of the muscles involved in BC was present after PS, together with a slowing of the CoP sway speed in both AP and ML directions. These results suggest that, despite the comparable CoP perimeter and area between PRE- and POST-PS, the BC was altered. Nevertheless, these effects on muscle activation lasted less than 15 min, while the CoP sway speed remained depressed for up to 30 min. A greater muscle activation could be an expression of two main mechanisms that may have occurred simultaneously. First, under a neuromuscular point of view, PS was found to reduce the motor drive toward a muscle (as witnessed by the present decrease in maximum force expression and sEMG RMS during MVC) *via* an alteration in the somatosensory feedback [4]. This alteration was initially supposed to act at both supraspinal (i.e., cortical and subcortical) and spinal levels [4]. However, more recent studies reported that cortical activation was unchanged after PS [26–29]. We can therefore speculate that PS might have altered the remaining supraspinal pathways at subcortical level (i.e., cerebellum, basal ganglia, and ventral-anterior and ventral-lateral thalamus nuclei) [30]. Since these play an important role in managing BC, it could be hypothesised that the PS-induced alteration in the sensorimotor network at subcortical level might lead to a less efficient BC, thus increasing muscle activation during BC tests. However, this remains to be supported. At spinal level, despite a recent study observing no PS-induced changes in H-reflex amplitude [26], it could be still hypothesised that stretching reduces the intrafusal muscle spindle discharge through muscle spindle desensitization, which cannot be detected by H-reflex changes [26, 28]. This alteration could slow down the reflex activation of the musculature responsible for BC, likely explaining the reduction in speed in the AP and ML directions found in the present study.

Second, under a mechanical point of view, the possible PS-induced reduction in muscle and/or muscle-tendon unit stiffness [5, 7] might have led to greater muscle activation to stabilize the joints involved in BC. This mechanism may also further explain the reduction in CoP sway speed observed after stretching. The different recovery time-course between muscle activation (<15 min) and CoP sway speed (>30 min) may suggest longer duration of the mechanical vs neuromuscular impairments. Indeed, previous studies reported that the PS-induced impairments in mechanical last more than the neuromuscular factors [31, 32].

The present results are partially in line with previous investigations, in which an overall worsening in BC after PS was reported [3, 8, 9]. In particular, an increase in postural sway accompanied by an increase in *gastrocnemius lateralis* activation was previously reported [3]. In the same study, the recovery of the two variables occurred within 10 min [3]. On the contrary, previous studies found an improvement in BC after stretching lower-limb muscles [10–12]. Interestingly, BC improved after 15 s PS in unexperienced participants, while no change was observed after 45 s [10]. This might suggest dose-dependent PS-induced effects on BC, and further studies are needed to better investigate the mechanisms underlying this possible phenomenon.

As for the static condition, PS-induced increase in BC muscle activation without any change of CoP sway area and perimeter was also found in dynamic balance test, suggesting comparable changes in neuromuscular control strategies for both static and dynamic BC. The present results are in line with the previous literature, in which PS did not affect BC during dynamic tests [10, 33, 34]. In contrast, one study retrieved an increase in dynamic balance tasks after PS [13]. A PS-induced decrease in muscle-tendon unit stiffness was advocated for the reduction in stretch-reflex activity, thus improving BC [13]. Nonetheless, this hypothesis needs to be further explored. Moreover, the elderly population involved in the previous study may partially explain the different outcomes.

A PS-induced increase in muscle activation without any change in CoP sway area and perimeter was found in all tests, regardless of the modalities in which they were conducted (i.e., OE, CE, +foam). Contrary to our hypothesis, this would suggest that the PS-induced alteration in the somatosensory feedback was not compensated by any intervention of the vestibular and visual systems. To the best of our knowledge, this is the first study evaluating the PS-induced effects on muscle activation and CoP sway parameters in such conditions, making the comparison with the literature difficult.

### Study limitations

The present study comes with some limitations. First, only the effect of PS was investigated. Other modalities, such as dynamic stretching or proprioceptive neuromuscular facilitation may yield to different results. Second, the study included young healthy subjects, limiting the generalization of the outcomes to other populations, e.g., elderly people. Further studies with different stretch modalities in different populations are therefore required. Third, the duration of the stretching session may not correspond to what usually practiced. Lastly, as done in previous study [35], a non-linear measure on the dynamics of the center of pressure was not performed due to limits of the device, and we acknowledge that this could have helped to deepen this aspect.

## Conclusion

The current study showed that PS did not affect the BC ability. However, greater commitment of the musculature responsible for BC was necessary to maintain similar levels of CoP adjustments. This phenomenon had a transient effect, as the increased activation recovered to pre-stretch levels within 15 min. This occurrence took place regardless of the condition in which BC was tested (static/dynamic, open/closed eye, with/without foam), suggesting that, at least in acute, compensatory mechanisms were not established with respect to the alteration of somatosensory feedback induced by PS. Altogether, this could imply a decrease in the neuromuscular efficiency when performing balance tasks after a PS bout.

In practice, PS could be safely used before performing both static and dynamic balance exercises. This may be considered when, as an example, people coming from previous injuries are requested to restore both the range of motion of a given joint and the balance ability in complex tasks. In sports context, the present results provide evidence that balance tasks might be preceded by PS, when necessary, without compromising the final performance.

## Supporting information

S1 Data (XLSX)

## Abbreviations

+foam: with foam pad
AP: anteroposterior
BC: balance control
CE: closed eyes
CoP: centre of pressure
CTRL: control session
ML: mediolateral
MVC: maximum voluntary isometric contraction
OE: open eyes
PS: passive stretching
RMS: root mean square
ROM: range of motion
sEMG: surface electromyography
STR: stretching session
